# Supplementary figures and images for: Distribution of Core Root Microbiota of Tibetan Hulless Barley along an Altitudinal and Geographical Gradient in the Tibetan Plateau
Source: Microorganisms. 2022 Aug 29;10(9):1737. doi: 10.3390/microorganisms10091737 (PMC9504843; doi:10.3390/microorganisms10091737)

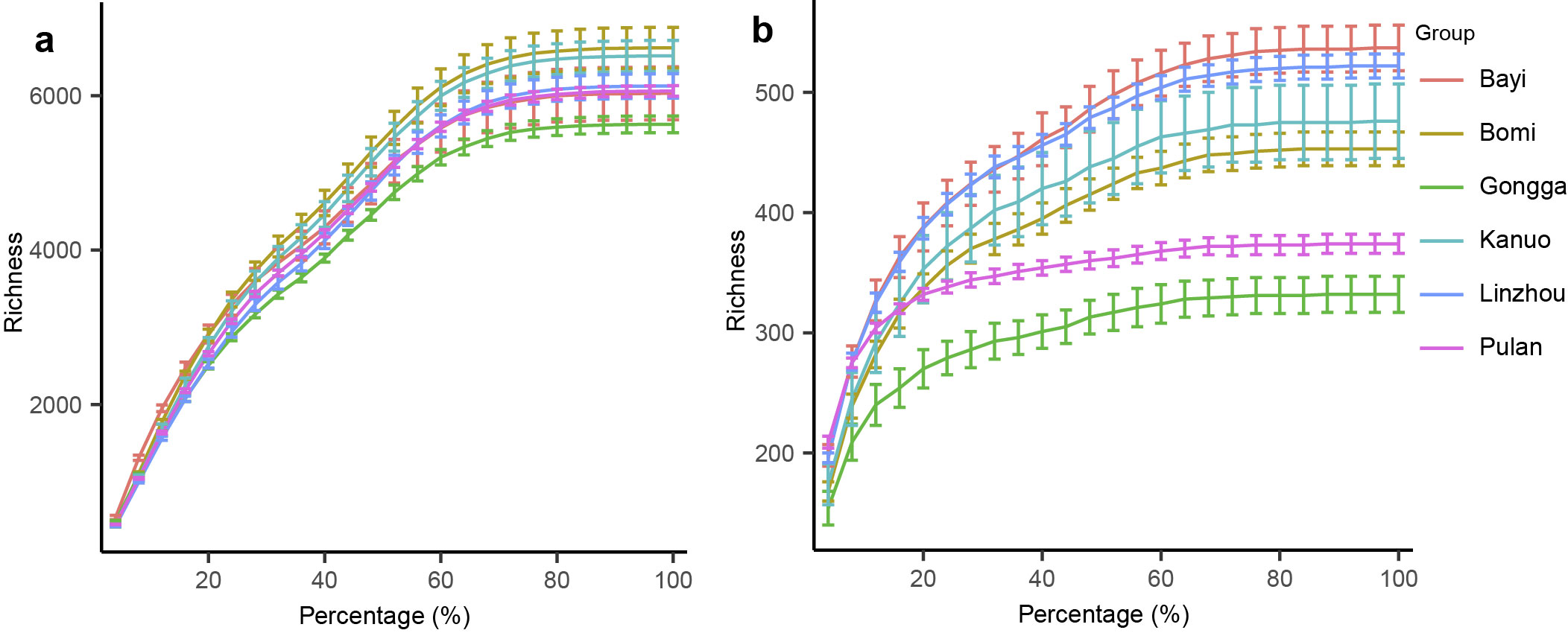

Supplement: Supplementary file 1 [file microorganisms-10-01737-s001.zip › Figure S1.jpg]

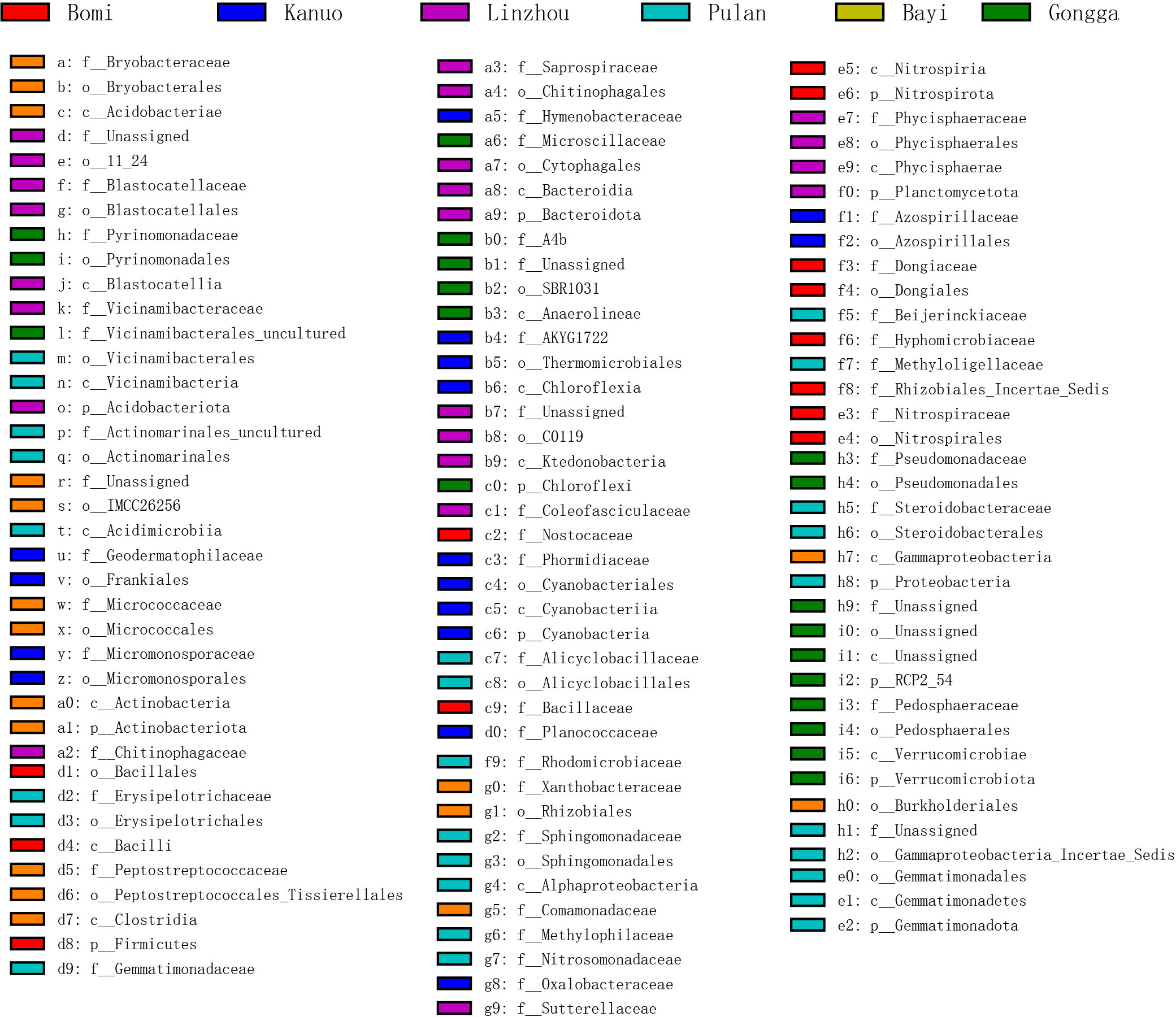

Supplement: Supplementary file 1 [file microorganisms-10-01737-s001.zip › Figure S2.jpg]

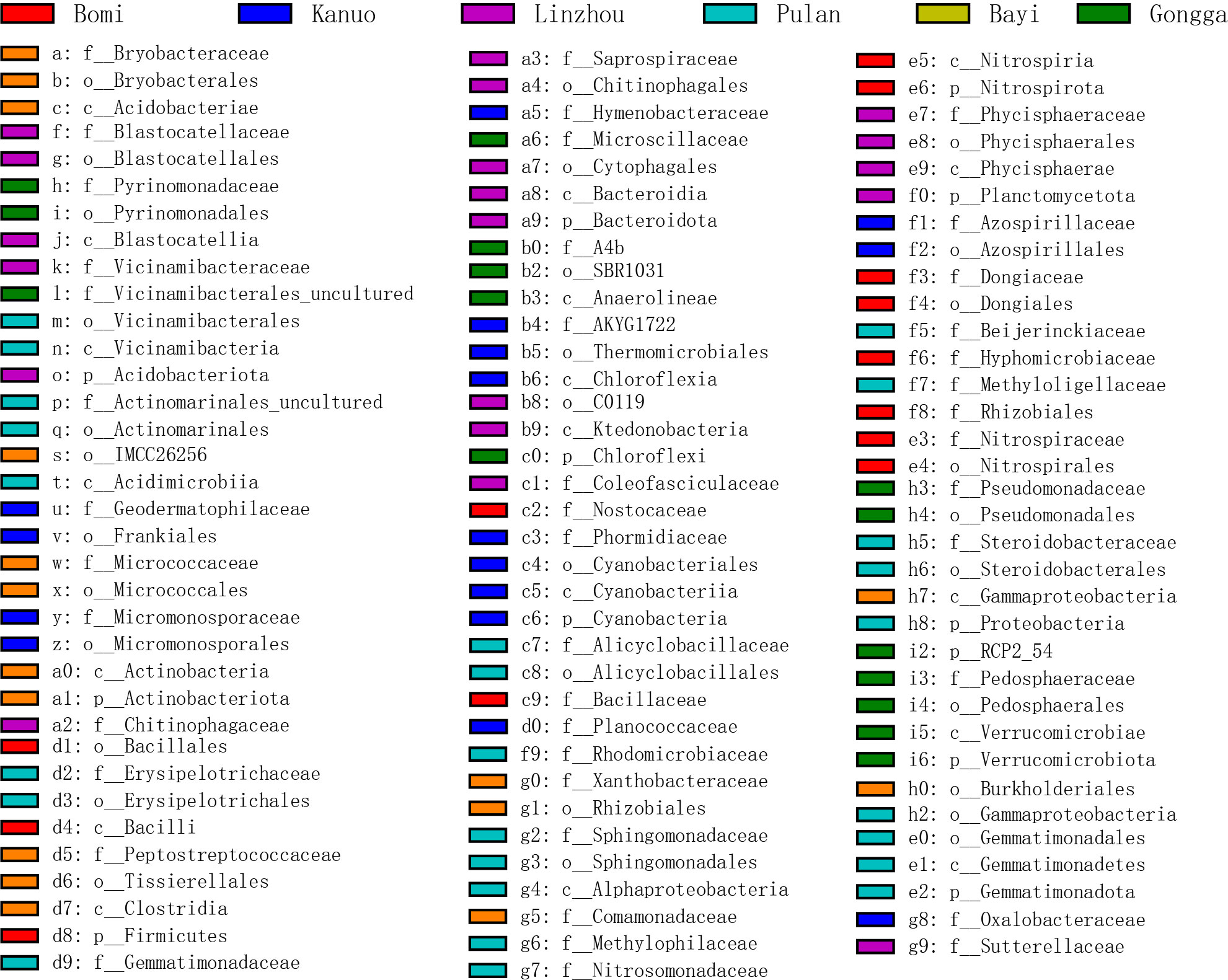

Supplement: Supplementary file 1 [file microorganisms-10-01737-s001.zip › Figure S3.jpg]

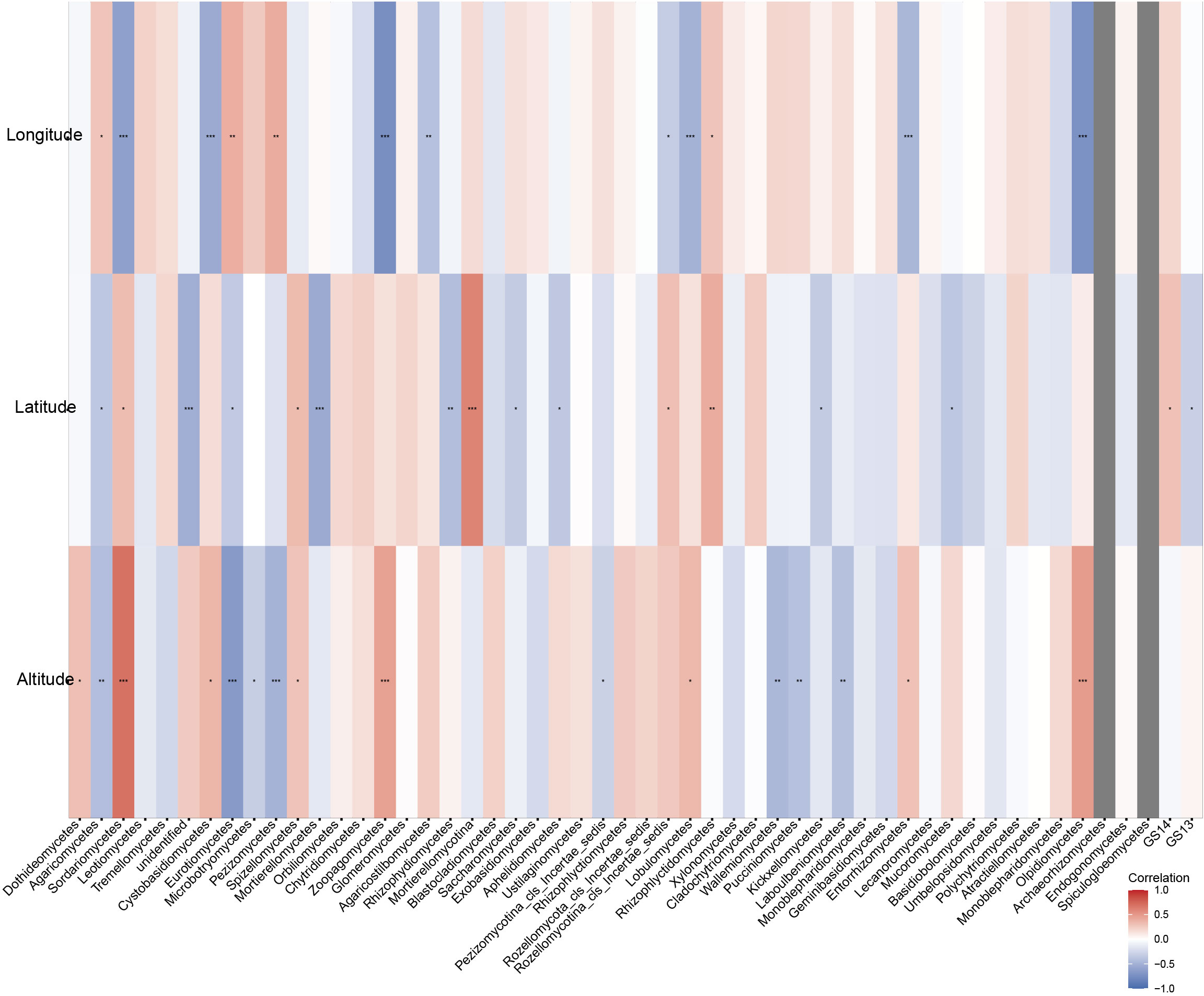

Supplement: Supplementary file 1 [file microorganisms-10-01737-s001.zip › Figure S4.jpg]

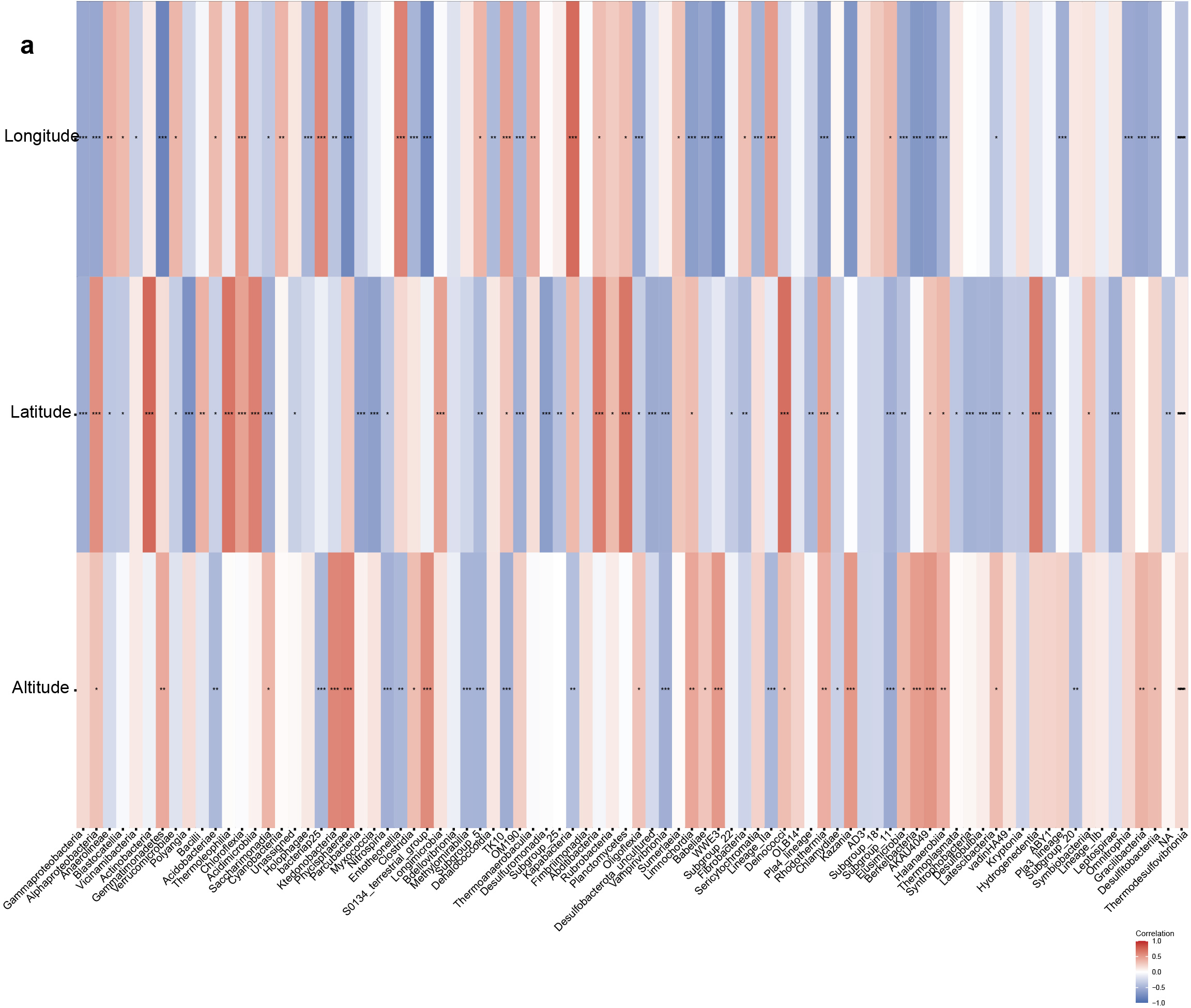

Supplement: Supplementary file 1 [file microorganisms-10-01737-s001.zip › Figure S5.jpg]

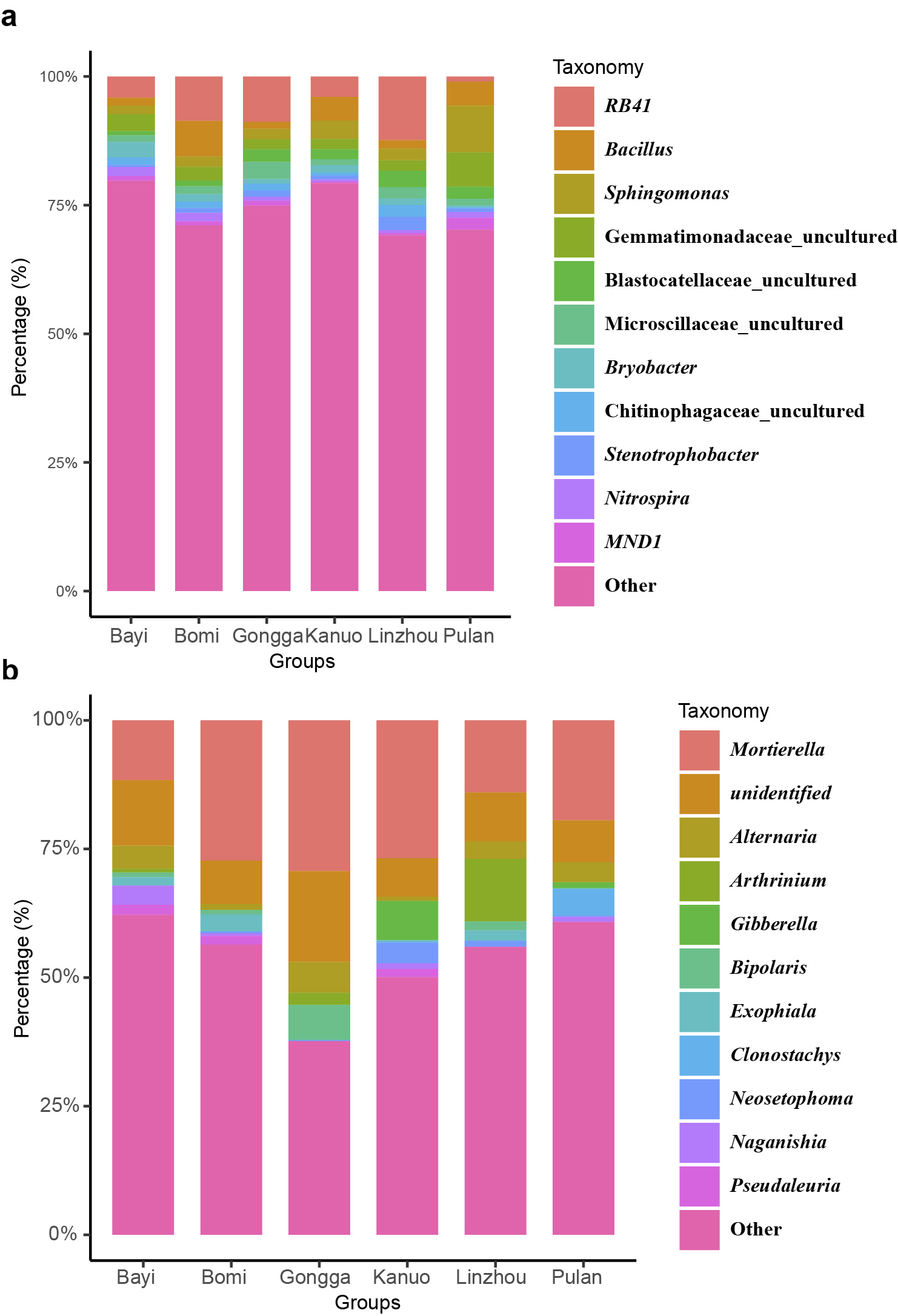

Supplement: Supplementary file 1 [file microorganisms-10-01737-s001.zip › Figure S6.jpg]
